# A novel financial risk assessment model for companies based on heterogeneous information and aggregated historical data

**DOI:** 10.1371/journal.pone.0208166

**Published:** 2018-12-26

**Authors:** Dan-Ping Li, Si-Jie Cheng, Peng-Fei Cheng, Jian-Qiang Wang, Hong-Yu Zhang

**Affiliations:** 1 School of Business, Hunan University of Science and Technology, Xiangtan, China; 2 School of Geosciences and Info-Physics, Central South University, Changsha, China; 3 Hunan Engineering Research Center for Intelligent Decision Making and Big Data on Industrial Development, Xiangtan, China; 4 School of Business, Central South University, Changsha, China; Shandong University of Science and Technology, CHINA

## Abstract

The financial risk not only affects the development of the company itself, but also affects the economic development of the whole society; therefore, the financial risk assessment of company is an important part. At present, numerous methods of financial risk assessment have been researched by scholars. However, most of the extant methods neither integrated fuzzy sets with quantitative analysis, nor took into account the historical data of the past few years. To settle these defects, this paper proposes a novel financial risk assessment model for companies based on heterogeneous multiple-criteria decision-making (MCDM) and historical data. Subjective and objective indexes are comprehensively taken into consideration in the financial risk assessment index system of the model, which combines fuzzy theory with quantitative data analysis. Moreover, the assessment information obtained from historical financial information of company, credit rating agency and decision makers, including crisp numbers, triangular fuzzy numbers and neutrosophic numbers. Furthermore, the Technique for Order of Preference by Similarity to Ideal Solution (TOPSIS) method is used to determine the ranking order of companies according to their financial risk. Finally, an empirical study of financial risk assessment for companies is conducted, and the results of comparative analysis and sensitivity analysis suggest that the proposed model can effectively and reliably obtain the company with the lowest financial risk.

## Introduction

Financial risk involves a combination of different methods, models and approaches to reduce the likelihood of a threat and the extent of losses [1]. Financial analysis can help to companies to detect financial risks in advance, take appropriate actions to minimize the losses, and support better decision-making [2, 3]. An accurate understanding and a well assessment of financial risk would have lots of positive consequences such as reduction of insolvency, reduction of bankruptcy rate, reduction of financial hardship. Therefore, the establishment of financial risk assessment model, the early diagnosis of the financial crisis and take appropriate measures to maintain health and safety and sustainable development of enterprises, it is very important [4]. Consequently, it’s necessary to study and develop an appropriate approach to assess financial risk of companies.

In the field of financial risk assessment, although some remarkable achievements have been made, there are still three shortcomings. First, the quantitative and qualitative analysis which adopts the combination of fuzzy theory and data analysis have not been used in financial risk assessment. Second, the historical data over several years have not been considered. Third, the information is used partially in financial risk assessment. The financial risk can be assessed by historical financial information of company, credit rating agency and decision makers. Hence, fuzziness and accuracy are existed simultaneously in the assessment information. The existing approaches only considered the data information of company, which may lead to information loss [5]. Therefore, in order to overcome these shortcomings, a novel financial risk assessment model for companies needs to be studied. To sum up, the motivations of this article are as follows:

1. The assessment of financial risk involves quantitative and qualitative indexes. Some scholars have employed objective financial indexes to assess financial risk quantitatively [6–9]. Subjective indexes such as controlling system of financial risk have not been utilized in extant study. Thus, the subjective indexes combined with objective indexes are employed in the index system of the proposed financial risk assessment model.
2. With respect to the partial use of information in assessment [10], it is appropriate to apply historical financial information and fuzzy theory to describe assessment information about financial risk for companies. The assessment information from historical financial information of company mainly involves crisp numbers. Wang et al. [11] presented that triangular fuzzy numbers can reflect the uncertainty of objective things and the fuzziness of human thought. Thus, it can be used to improve the objectivity and accuracy of the description of credit rating. Zhang et al. [12] presented that a neutrosophic set is an effective tool for reflecting the fuzziness in text evaluation because the evaluation information from decision makers is text information that represents sentiment values, and every sentiment value has not only a certain degree of truth, but also a falsity degree and an indeterminacy degree [13]. Thus, it needs to transform sentiment values into neutrosophic numbers with positive, medium, and passive values. For example, when asked to assess whether controlling system of financial risk would be “good”, from the sentiment value of a decision maker, we may deduce that the membership degree of truth is 0.8, the membership degree of indeterminacy is 0.1, and the membership degree of falsity is 0.1. Therefore, assessment information, including crisp numbers, triangular fuzzy numbers and neutrosophic numbers, needs to be taken into account in the financial risk assessment model.
3. In order to deal with the ranking order of companies according to their financial risk based on historical data and heterogeneous MCDM, a systematic approach need to be employed in the proposed model. Shih et al. [14] pointed out that TOPSIS is a practical and useful technique for the ranking and selection of a number of externally determined alternatives through distance measures, and it has been applied in multiple-criteria decision-making (MCDM) [15]. Lourenzutti et al. [16] and Li et al. [17] proposed heterogeneous TOPSIS for multi-criteria decision making method. Therefore, the TOPSIS method is used to obtain the ranking order of companies in the financial risk assessment model.

In this paper, a novel financial risk assessment model is developed to help managers assess company’s financial risk by utilizing TOPSIS according to above discussion, which is based on MCDM and heterogeneous information including qualitative data and non-qualitative data. The contributions of this paper are concluded as three aspects. The first one is the establishment of an improved financial risk index system with comprehensive consideration of subjective and objective indexes, which combines fuzzy theory with quantitative data analysis. The second is the consideration of the impact of historical financial position on current financial risk analysis by aggregating historical data over several years which assesses financial risk accurately. The third is the application of heterogeneous information obtained from historical financial information, credit rating and decision makers, including crisp numbers, triangular fuzzy numbers and neutrosophic numbers. The final contribution is that the TOPSIS method for heterogeneous multi-criteria decision-making is employed to get the ranking order of companies based on their financial risk, which can help manager to assess financial risk.

The rest of the paper is organized as follows. In Section 2, previous researches about financial risk assessment are introduced briefly. A brief introduction about research methodology including TOPSIS method, exponential smoothing method, neutrosophic number and triangular fuzzy number is presented in Section 3. Subsequently, a novel financial risk assessment model is developed based on heterogeneous MCDM in Section 4. In Section 5, an empirical study is presented concretely, and the effectiveness of the proposed method is verified by a comparative analysis. Finally, Section 6 summarizes the paper and proposes some directions for future research.

## Literature review

Among the studies of financial risk of listing companies, numerous scholars have made great contributions. Some scholars evaluated financial risk by utilizing quantitative analysis. For example, the utility functions that classify the considered alternatives into predefined risk classes were developed in the study by Doumpos and Zopounidis [6]. It proposed the multi-group hierarchical discrimination method (M.H.DIS) that classified countries into four groups like *c*_*1*_, *c*_*2*_, *c*_*3*_, *c*_*4*_ from good to bad. Lee et al. [7] evaluated financial positions of shipping companies using entropy and grey relation analysis. Stochastic frontier analysis (SFA) was illustrated in the study proposed by Wang et al. [18], which calculated efficiency estimation of risk indicators with determined influence factors of risk assessment indicators computed by panel frontier model. Financial ratios of capital structure risk, liquidity risk and insolvency risk studied by balance sheet, statement of income, expenses and cash flow of dozens of businesses were described to assess financial risk in Kociu et al. [8]. Furthermore, many fuzzy theory and MCDM method have been applied in business [19] and risk assessment. Sabokbar et al. [20], Mardani et al. [21] and Ribeiro et al. [22] utilized fuzzy set to assess risk. Kochanek and Tynan [23] adopted linguistic label to present uncertainty of risk. Chang et al. [24] adopted the Fuzzy Analytic Network Process (FANP) method to assess ERP implementation risks. Gonçalves et al. [25] employed the Interactive Multiple Criteria Decision Making (TODIM) approach to analyze credit risk. Kou et al. [26] presented an MCDM to analyze financial risk. Shaverdi et al. [27] ranked companies by using fuzzy AHP and fuzzy TOPSIS comparatively to evaluate financial performance. In conclusion, in the extant researches, fuzzy theory and quantitative analysis have not been employed simultaneously to assess financial risk.

With respect to financial risk index system, numerous assessment indexes have been researched. Cui et al. [28] established a financial evaluation index system of Chinese listing companies with four financial risk criteria and corresponding objective numerical indexes, including financing risk, investment risk, income distribution risk and cash flow at risk. The example for country risk assessment conducted by Doumpos and Zopounidis [6] used twelve economic indicators like import and export volume growth and GNP growth as risk evaluation indexes. Jurczyk et al. [29] quantified systemic risks by numerous stock indexes, and without non-numerical indexes. Wang and Liu [30] evaluated the real estate investment risk by qualitatively analyzing financing risk and investment site risk, etc. Gonçalves et al. [25] used fuzzy theory to analyze credit risk. In view of the above-mentioned review, a financial risk index system including quantitative and qualitative index needs to be researched.

As studied in numerous risk evaluation researches, existing approaches used partial information. Wang and Liu [30] utilized crisp numbers to evaluate financing risk qualitatively. Kiliçman and Sivalingam [31] used triangular fuzzy numbers to represent return rates, etc. Kochanek and Tynan [23] adopted linguistic label to present uncertainty of risk. And every linguistic value has not only a certain degree of truth, but also a falsity degree and an indeterminacy degree; it needs to transform sentiment values into neutrosophic numbers with positive, medium, and passive values. None of the previous risk evaluation research considered these information types mentioned above simultaneously. Thus, in this paper, the evaluation information obtained from historical financial information, credit rating agency and decision makers includes crisp numbers, triangular fuzzy numbers and neutrosophic numbers, which need to be considered in the assessment progress.

In current methods of subjective weight, numerous scholars have made notable contributions. Wang et al. [32] used criteria priorities to compute criteria weights. Zhao et al. [33] calculated weight by using a probabilistic method. Mangla et al. [34] used fuzzy analytic hierarchy process (AHP) to get subjective weight or provided by decision-makers. Rezaei [35, 36] utilized the (best-worst method) BWM method to calculate subjective weight with lesser comparison times and information loss compared with AHP. Tian et al. [37] presented that the BWM method can require fewer pairwise comparisons than does fuzzy AHP but obtain more highly reliable weights. Therefore, the subjective weight of financial criteria in the proposed model is calculated by BWM.

In conclusion, previous researches about financial risk evaluation should be modified in future study. In order to settle these issues based on the above discussion, we (1) establish a novel financial risk index system combining fuzzy theory with quantitative analysis, (2) consider various types of information including crisp numbers, triangular fuzzy numbers and neutrosophic numbers, (3) utilize BWM method to calculate the subjective weight of financial risk criteria, (4) utilize TOPSIS method to manage heterogeneous information and obtain the ranking order of companies according to their financial risk.

## Research methodology

In this section, the specific and processes of TOPSIS method, the concepts, definitions and algorithms of neutrosophic set and triangular fuzzy number are introduced.

### TOPSIS method

The TOPSIS method, an MCDM method, was proposed by Hwang and Yoon in 1981 [38]. It provides the best alternative which is as close as possible to the best solution. Dozens of scholars have applied TOPSIS to solve simple or complex problems in different areas [39], e.g., weapon selection, alternative evaluation and risk assessment [40–42]. The procedure of the TOPSIS method can be described as shown in the following steps [16]:

Step 1 Define and normalize the decision matrix *R* = (*r*_*ij*_).
Step 2 Aggregate the weights to the decision matrix by making *v*_*ij*_ = *w*_*j*_*r*_*ij*_.
Step 3 Define the positive ideal solution (PIS), $v_{j}^{+}$, and the negative ideal solution (NIS), $v_{j}^{-}$, for each criterion. Usually, $v_{j}^{+}=\text{max}\left\{v_{ij},\ldots ,v_{mj}\right\}$ and $v_{j}^{-}=\text{min}\left\{v_{ij},\ldots ,v_{mj}\right\}$ for benefit criteria, and $v_{j}^{+}=\text{min}\left\{v_{ij},\ldots ,v_{mj}\right\}$ and $v_{j}^{-}=\text{max}\left\{v_{ij},\ldots ,v_{mj}\right\}$ for cost criteria.
Step 4 Calculate the separation measures for each alternative.
  $$S_{i}^{+}=\sqrt{\sum_{j=1}^{n}{\left(v_{j}^{+}-v_{ij}\right)}^{2}},\;\;\;\;i=1,2,\ldots ,m$$ (1)
  $$S_{i}^{-}=\sqrt{\sum_{j=1}^{n}{\left(v_{j}^{-}-v_{ij}\right)}^{2}},\;\;\;\;i=1,2,\ldots ,m.$$ (2)
Step 5 Calculate the closeness coefficients to the ideal solution for each alternative.
  $$CC_{i}=\frac{S_{i}^{-}}{S_{i}^{-}+S_{i}^{+}}.$$ (3)
Step 6 Rank the alternatives according to *CC*_*i*_. The bigger *CC*_*i*_ is, the better alternative *A*_*i*_ will be.

### Exponential smoothing method

First, the trend changes of time series can be distinguished based on data visualization by using professional software. Then, the procedure of the method is given as follows:

$$\begin{array}{l} S_{t+1}=\partial X_{t}+(1-\partial )S_{t}\\ \partial \in [0,1] \end{array}.$$ (4)

Let *∂* is weighting coefficient which represents the weight of latest data (the bigger *∂* is, the more important the new data is). *S*_*t*+1_ is the (t+1)th prediction value, *X*_*t*_ is the *t* phase data. The most important aspect of exponential smoothing is the solution of *∂* and original value, as follows.

If the time series is stable comparatively, the selection of *∂* is a lower value as (0.1–0.3); on the contrary, it may bigger like (0.6–0.8). Different *∂* value is selected subjectively, count mean absolute error (MAE) with different *∂* like formula (4), the *∂* value is best which minimize the error. For *s*_*i*_ is predicted value, *x*_*i*_ is true value, the solution is depicted as formula (5).

$$MAE=\frac{1}{n}\sum_{i=1}^{n}\left|s_{i}-x_{i}\right|.$$ (5)

Let *S*_0_ is the original value; formula (6) can describe the definite way of *S*_0_. When *t* < 20, in general, *S*_0_ is the mean value of three years’ true data initially, in this article we elicit two stages.

$$S_{0}=\{\begin{array}{ll} {\text{x}}_{1}, & t\geq 20\\ \frac{x_{1}+x_{2}+x_{3}}{3}, & t<20 \end{array}.$$ (6)

### Neutrosophic set theory

**Definition 1**. [43] Let *X* be a space of points (objects), with a generic element in *X* denoted by *x*. Then an NS *A* in *X* is characterized by three membership functions, including a truth-membership function *T*_*A*_(*x*), indeterminacy-membership function *I*_*A*_(*x*), and falsity-membership function *F*_*A*_(*x*), and is defined as‬‬‬‬‬‬‬‬‬‬‬‬‬‬‬‬‬‬‬‬‬‬‬‬‬‬‬ *A =* {˂*x*, *T*_*A*_(*x*), *I*_*A*_(*x*), *F*_*A*_(*x*) ˃ | *x*∈*X*}, where *T*_*A*_(*x*), *I*_*A*_(*x*), and *F*_*A*_(*x*) are real standard or nonstandard subsets of ]-0, 1+[, i.e. *T*_*A*_(*x*): *X* →]-0, 1+[, *I*_*A*_(*x*): X →]-0, 1+[, *F*_*A*_(*x*): *X* →]-0, 1+[. The sum of *T*_*A*_(*x*), *I*_*A*_*(x)*, and *F*_*A*_(*x*) is unrestricted, and *-0*≤ *T*_*A*_(*x*)+*I*_*A*_(*x*)+ *F*_*A*_(*x*) ≤ 3+.

**Definition 2**. [44] Let *X* be a universal space of points (objects), with a generic element of *X* denoted by *x*. A single-valued neutrosophic set (SVNS) $\tilde{N}\subset X$ is characterized by a truth membership $T_{\tilde{N}}(x)$, an indeterminacy-membership function $I_{\tilde{N}}(x)$ and a falsity-membership function $F_{\tilde{N}}(x)$ with $T_{\tilde{N}}(x)$, $I_{\tilde{N}}(x)$, $F_{\tilde{N}}(x)\in [0,1]$ for all *x*∈ *X*. The sum of three membership functions of a SVNS $\tilde{N}$, the relation $0\leq T_{\tilde{N}}(\text{x})+I_{\tilde{N}}(x)+F_{\tilde{N}}(x)\leq 3$ for all *x*∈ *X* holds good.

**Definition 3**. (Euclidean distance) [45] Let $\tilde{A}=\{\left(x_{1}|\langle T_{\tilde{A}}(x_{1}),I_{\tilde{A}}(x_{1}),F_{\tilde{A}}(x_{1})\rangle \right),\ldots ,\left(x_{n}|\langle T_{\tilde{A}}(x_{n}),I_{\tilde{A}}(x_{n}),F_{\tilde{A}}(x_{n})\rangle \right)\}$ and $\tilde{B}=\{\left(x_{1}|\langle T_{\tilde{B}}(x_{1}),I_{\tilde{B}}(x_{1}),F_{\tilde{B}}(x_{1})\rangle \right),\ldots ,\left(x_{n}|\langle T_{\tilde{B}}(x_{n}),I_{\tilde{B}}(x_{n}),F_{\tilde{B}}(x_{n})\rangle \right)\}$ be two SVNSs for *x*_*i*_ ∈*X (i = 1*, *2*, …, *n)*. Then the Euclidean distance between two SVNSs $\tilde{A}$ and $\tilde{B}$ can be defined as follows:

$$D(\tilde{A},\tilde{B})=\sqrt{\frac{1}{3n}\sum_{i=1}^{n}\{{\left(T_{\tilde{A}}(x_{i})-T_{\tilde{B}}(x_{i})\right)}^{2}+{\left(I_{\tilde{A}}(x_{1})-I_{\tilde{B}}(x_{1})\right)}^{2}+{\left(F_{\tilde{A}}(x_{1})-F_{\tilde{B}}(x_{1})\right)}^{2}\}}\;.$$ (7)

**Definition 4**. According to the study by Majumdar and Samanta [45], single-valued neutrosophic set *A =* {*˂x*, *T*_*A*_(*x*), *I*_*A*_(*x*), *F*_*A*_(*x*)˃|| *x*∈*X*}, an entropy on neutrosophic set *A* is computed as formula (8).

$$E(A)=1-\frac{1}{n}\sum_{x_{i}\in X}(T_{A}(x_{i})+F_{A}(x_{i}))⊗\left|I_{A}(x_{i})-I_{A^{c}}(x_{i})\right|.$$ (8)

**Definition 5**. The entropy weight of neutrosophic set in the study proposed by Tan et al. [46] is as follows:

$$W_{j}=\left(1-E\left(x_{j}\right)\right)/\sum_{j}^{n}\left(1-E\left(x_{j}\right)\right).$$ (9)

**Definition 6**. The single valued neutrosophic weighted averaging (SVNWA) aggregation operator proposed by Ye’s study [47] is as follows:

$$\begin{array}{ll} F_{A_{i}} & =\psi_{1}A_{1}⊕\psi_{2}A_{2}⊕\ldots ⊕\psi_{n}A_{n}\\ & =\langle 1-\prod_{i=1}^{n}{\left(1-T_{A_{i}}\right)}^{\psi_{i}},\prod_{k=1}^{n}{\left(I_{A_{i}}\right)}^{\psi_{i}},\prod_{i=1}^{n}{\left(F_{A_{i}}\right)}^{\psi_{i}}\rangle \end{array},$$ (10)

where *Ψ* = (*Ψ*_1_,*Ψ*_2_,…,*Ψ*_*n*_)^*T*^ is the weight vector of *A*_*i*_.

**Definition 7**. Suppose that *S* = {*s*_*i*_|*i* = −*t*,…,*t*} is a limited and ordered discrete label set [48]. In this system, we let *t* = 3, *s*_*i*_ represents a possible linguistic term. The specific label set could be: *S =* {*s*_*-3*_ = very bad, *s*_*-2*_ = bad, *s*_*-1*_ = slightly bad, *s*_*0*_ = ok, *s*_*1*_ = slightly good, *s*_*2*_ = good, *s*_*3*_ = very good}. The semantic values proposed in Table 1 are calculated throughout sentiment analysis by using the software of ‘The R Project for Statistical Computing’. According to different sentiment word, we allocate linguistic variable *s*_*i*_ to positive, neutral and passive value as a neutrosophic number *A*_*i*_ = <*T*_*i*_, *I*_*i*_, *F*_*i*_>, *i* = 1, …, *n*, (positive value is T value, neutral value is I, passive value is F). The T-value is the average value of positive values, while the I-value is 1 if s_0_ exists or 0 if s_0_ not exists, the F-value is mean of the absolute passive values. For example, a set *S* = {*s*_−1_, *s*_0_, *s*_1_, *s*_2_}, the neutrosophic value is *$\langle \frac{s_{1}+s_{2}}{2},s_{0},\left|s_{-1}\right|\rangle$*, i.e. $\langle \frac{0.106066+0.75}{2},1,0.10607\rangle$.

**Table 1:** Sentiment value.

Evaluation	Very Bad	Bad	Slightly Bad	OK	Slightly Good	Good	Very Good
Sentiment degree	-0.95459	-0.75	-0.10607	0	0.106066	0.75	0.954594

### Triangular fuzzy number

**Definition 8**. A fuzzy set $\tilde{a}$ is a triangular fuzzy number (TrFN) [49], denoted by ***a*** = (*a*_*1*_, *a*_*2*_, *a*_*3*_), if it is defined on the real line with membership function given by:

$$\mu_{\tilde{a}}(x)=\{\begin{array}{ll} \frac{x-a_{1}}{a_{2}-a_{1}} & a_{1}<x\leq a_{2}\\ \frac{a_{3}-x}{a_{3}-a_{2}} & a_{2}<x\leq a_{3}\\ 0 & otherwise \end{array},$$ (11)

where *a*_1_ < *a*_2_ < *a*_3_, *a*_1_ and *a*_2_ stand for the lower and upper values of the support of $\tilde{a}$, respectively, and *a*_2_ for the modal value.

**Definition 9**. Let *$\tilde{a}=(a_{1},a_{2},a_{3})$* and *$\tilde{\text{b}}=(b_{1},b_{2},b_{3})$* be two TrFN. A distance measure between *$\tilde{a}$* and $\tilde{\text{b}}$ is given in the study by Dağdeviren et al. [40]:

$$d(\tilde{a},\tilde{b})=\sqrt{\frac{1}{3}\sum_{i=1}^{3}(a_{i}-b_{i}{)}^{2}}.$$ (12)

**Definition 10**. A triangular fuzzy number is denoted by $\tilde{a}=\;\left(a_{1},\;a_{2},\;a_{3}\right)$, an interval value is denoted by *l* = [*a*,*b*]. Laarhoven and Pedrycz [50] indicated *a*_1_ and *a*_3_ is the upper limit value and lower limit value of triangular fuzzy number, and *a*_2_ is (*a*_1_ + *a*_3_)/2 when the TrFN $\tilde{a}$ is symmetrical. The lower value *a*_1_ and the upper value *a*_3_ are determined by standardized *a* and *b* respectively based on interval value [*a*, *b*]. Based on the definition of triangular fuzzy number, the value of *a*_2_ whose membership is 1 is the median value like $\frac{a+b}{2}$ in the situation of the shape of its membership function is an isosceles triangle. However, in the situation of irregular triangle, the value of *a*_2_ whose membership is 1 can be computed by following formula (13):

$$a_{2}=(1-\lambda )a+\lambda b,\;\;\;\;\lambda \in [0,1],$$ (13)

where *λ* represents attitude of evaluator. Value of *λ* = 1 indicates supportive attitude, *λ* = 0.5 indicates neutral attitude and *λ* = 0 indicates opposing attitude. As the evaluation of credit rating is determined by credit rating agency’s comprehensive assessment, we calculate *a*_2_ with *λ* = 0.5 i.e. $a_{2}=\frac{a+b}{2}$, the membership shape of this TrFN is an isosceles triangle. Then, the triangular fuzzy number can be determined by standardized interval value, for example, [70,80] can be transformed into triangular fuzzy number (0.7,0.75,0.8).

## Financial risk assessment model

The proposed financial risk assessment model consists five parts, as depicted in S1 Fig. The first part is the establishment of financial risk index system, the important criteria as well as sub-criteria are determined from literature reviews and experts. The second part is the calculation of subjective criteria weight by using BWM method. Moreover, the evaluation information from historical financial information of companies, credit rating agency and decision makers can be obtained. And the evaluation matrix is established including crisp numbers, triangular fuzzy numbers and neutrosophic numbers. In addition, with the computation of objective entropy weight of financial index, the comprehensive index weight can be calculated by multiplying entropy weight and subjective criteria weight. Finally, the ranking order of companies according to financial risk is derived utilizing TOPSIS method based on heterogeneous MCDM. The specific details of this novel model will be described in the rest of this section.

### The establishment of the financial risk index system

The assessment of financial risk involves financial condition of company, credit rating and evaluation of decision maker, and it is very complicated. Based on the discussion in the literature review, financial risk can be mainly evaluated from four criteria, which are financing risk, investment risk, income distribution risk and cash flow at risk, denoted as *A*_*i*_(*i* = 1, 2, 3, 4) respectively. Moreover, every criterion can be divided into multiple sub-criteria (i.e. financial indexes). Based on the definition of financial risk and previous studies analyzed in the literature review, fuzzy information is taken into account in the financial risk assessment model. Generally, the credit rating indicates the capacity of financing; the contractual capacity of partners represents the fund risk; and the management system of financial risk indicates the risk management ability of company. Therefore, the credit rating index is added to financing risk criterion, and the contractual capacity of partner index and the controlling system of financial risk index are added to investment risk criterion. Hence, an improved risk index system is established as shown in S2 Fig. Because of the complexity of financial condition and the uncertainty of information, the assessment values of financial risk indexes can be divided into multiple types. Therefore, heterogeneous information including crisp numbers, triangular fuzzy numbers and neutrosophic numbers exists in this proposed financial risk index system. The financial risk index system including the definition of the financial risk indexes is established in Table 2.

**Table 2:** Definition of criteria and indexes.

Criteria	Indexes	Definition	Index Type
Financing risk (A_1_)	Asset liability ratio(*a*_*11*_)	The rate of liabilities to assets	Cost
	Current ratio(*a*_*12*_)	The ratio measures how many times the current assets are compared to the current liabilities.	Benefit
	Quick ratio(*a*_*13*_)	The ratio means the ability of quick assets extinguishing current liabilities.	Benefit
	Number of times interest earned(*a*_*14*_)	The rate of earnings before interest and tax to interest expense	Benefit
	Credit rating(*a*_*15*_)	A judgment about a business’s credit standing	Benefit
Investment risk(A_2_)	Main business cost ratio(*a*_*21*_)	The rate of main business cost to main business income	Cost
	Operating expense ratio(*a*_*22*_)	The scale of operating expenses in the operating revenue	Cost
	Main business revenue growth rate(*a*_*23*_)	The ratio measures the range of main business revenue growth	Benefit
	Total asset growth rate(*a*_*24*_)	The range of total asset growth	Benefit
	Net assets yield *a*_*25*_	The rate of after-tax profits to ownership interest	Benefit
	Net profit growth rate(*a*_*26*_)	The increasing range of net profit	Benefit
	Contractual capacity of partner(*a*_*27*_)	The index means the ability of fulfilling contracts. The stronger the ability is, the lower the default risk is.	Benefit
	Controlling system of financial risk(*a*_*28*_)	It presents the ability of financial risk management.	Benefit
Income distribution risk(A_3_)	Shareholder’s equity growth rate(*a*_*31*_)	The growth range of shareholder’s equity	Benefit
	Equity ratio(*a*_*32*_)	The rate indicates the relative proportion of equity used to finance a company’s assets.	Benefit
	Retention ratio(*a*_*33*_)	The percentage of earnings belong to retained earnings	Benefit
Cash flow at risk(A_4_)	Cash debt coverage ratio(*a*_*41*_)	The capacity of cash that could settle the liability	Benefit
	Cash ratio(*a*_*42*_)	The rate expresses the relationship of cash and cash equivalents to the current liabilities.	Benefit
	Security surplus cash multiples(*a*_*43*_)	The rate of net operating cash flow to net profit	Benefit

### The estimation of criteria weights with BWM

According to the discussion in the literature review, a more efficient method (i.e. BWM method) is used to calculate the subjective weight in this section. The detailed steps of BWM to compute the weights of the four financial risk criteria are summarized as follows [35].

Step 1 Determine a set of decision criteria.
  In this step, we consider the criteria {*c*_1_, *c*_2_, …, *c*_*n*_} that should be used to arrive at a decision.
Step 2 Determine the best (e.g. most desirable, most important) and the worst (e.g. least desirable, least important) criterion.
Step 3 Determine the preference of the best criterion over all the other criteria, using a number between 1 and 9. The resulting best-to-others vector would be: *A*_*B*_ = (*a*_*B*1_, *a*_*B*2_, …, *a*_*Bn*_) where *a*_*Bj*_ indicates the preference of the best criterion B over criterion *j*. It is clear that *a*_*BB*_ = 1.
Step 4 Determine the preference of all the criteria over the worst criterion, using a number between 1 and 9. The resulting others-to-worst vector would be: *A*_*W*_ = (*a*_1*W*_, *a*_2*W*_, …, *a*_*nW*_) where *a*_*jW*_ indicates the preference of the criterion *j* over the worst criterion W. It is clear that *a*_*WW*_ = 1.
Step 5 Find the optimal weights $\left(w_{1}^{*},w_{2}^{*},\ldots ,w_{n}^{*}\right)$.

The optimal weight for the criteria is the one where, for each pair of *w*_*B*_ / *w*_*j*_ and *w*_*j*_ / *w*_*W*_, we have *w*_*B*_ / *w*_*j*_ = *a*_*Bj*_ and *w*_*j*_ / *w*_*W*_ = *a*_*jW*_. To satisfy these conditions for all *j*, we should find a solution where the maximum absolute differences $\left|\frac{w_{B}}{w_{j}}-a_{Bj}\right|$ and $\left|\frac{w_{j}}{w_{w}}-a_{jw}\right|$ for all *j* is minimized. Considering the non-negativity and condition for the weights, the following problem is resulted:

$$\begin{array}{l} \text{min}\;{\text{max}}_{j}\{\left|\frac{w_{B}}{w_{j}}-a_{Bj}\right|,\left|\frac{w_{j}}{w_{w}}-a_{jw}\right|\}\\ s.t.\\ \sum_{j}w_{j}=1\\ w_{j}\geq 0,\text{for}\;all\;j \end{array}.$$ (14)

The formula (14) is equivalent to the following formula:

$$\begin{array}{l} \text{min}\;\xi \\ s.t.\\ \left|\frac{w_{B}}{w_{j}}-a_{Bj}\right|\leq \xi ,for\;all\;j\\ \left|\frac{w_{j}}{w_{w}}-a_{jw}\right|\leq \xi ,for\;all\;j\\ \sum_{j}w_{j}=1\\ w_{j}\geq 0,\text{for}\;all\;j \end{array}.$$ (15)

Solving problem (15), the optimal weights $\left(w_{1}^{*},w_{2}^{*},\ldots ,w_{n}^{*}\right)$ and ξ* are obtained.

Then calculate the consistency ratio, using ξ*and the corresponding consistency index, as follows:

$$\begin{array}{l} Consistency\;Ratio \end{array}=\frac{\xi^{*}}{Consistency\;Index}.$$ (16)

Table 3 shows the maximum values of *ξ* (consistency index) for different values of *a*_*BW*_ [35].

**Table 3:** Consistency index (CI).

*a*_*Bw*_	1	2	3	4	5	6	7	8	9
Consistency Index(max *ξ*)	0.00	0.44	1.00	1.63	2.30	3.00	3.73	4.47	5.23

If consistency ratio ≤ 0.1, it implies a very good consistency which is acceptable. Otherwise we can revise *a*_*Bj*_ and *a*_*jW*_ to make the solution (more) consistent.

BWM introduced above is limited to derive unique optimum weight vector when the number of criteria is more than three. It might leads to multiple optimal solutions. The improved method present in [36] is used to obtain optimal weights with *n* criteria. If we use {|w_*B*_ − *a*_*Bj*_*w*_*j*_|, |*w*_*j*_ − *a*_*jw*_*w*_*w*_|} instead of $\{\left|\frac{w_{B}}{w_{j}}-a_{Bj}\right|,\left|\frac{w_{j}}{w_{w}}-a_{jw}\right|\}$, the problem can be solved as follows.

$$\begin{array}{l} \text{min}\;{\text{max}}_{j}\{\left|{\text{w}}_{B}-a_{Bj}w_{j}\right|,\left|w_{j}-a_{jw}w_{w}\right|\}\\ s.t.\\ \sum_{j}w_{j}=1\\ w_{j}\geq 0,\;for\;\text{all}\;j \end{array}.$$ (17)

The formula (17) can be transferred to the following linear programming problem:

$$\begin{array}{l} \text{min}\;\xi^{L}\\ s.t.\\ \left|w_{B}-a_{Bj}w_{j}\right|\leq \xi^{L},for\;\text{all}\;j\\ \left|w_{j}-a_{jw}w_{w}\right|\leq \xi^{L},for\;all\;j\\ \sum_{j}w_{j}=1\\ w_{j}\geq 0,for\;all\;j \end{array}.$$ (18)

Formula (18) is a linear problem, which can calculate the only optimal weights $\left(w_{1}^{*},w_{2}^{*},\ldots ,w_{n}^{*}\right)$. Hence we can compute the weight vector (*w*_1_, *w*_2_, *w*_3_, *w*_4_) of financing risk *A*_1_, investment risk *A*_2_, income distribution risk *A*_3_ and cash flow at risk *A*_4_.

### The evaluation matrix of financial risk

The financial risk evaluation of company involves qualitative and quantitative indicators such as financial status, credit rating, decision makers, etc. Different decision makers may make different assessment based on their distinct knowledge and different judgment standards. Therefore, in this section, the evaluation information determined by historical financial information, credit rating and decision makers is heterogeneous, including crisp numbers, interval numbers and linguistic labels. Specifically, the crisp numbers are the evaluation values of asset liability ratio, current ratio, quick ratio, number of times interest earned, main business cost ratio, operating expense ratio, main business revenue growth rate, total asset growth rate, net assets yield, net profit growth rate, shareholder’s equity growth rate, equity ratio, retention ratio, cash debt coverage ratio, cash ratio and security surplus cash multiples; the interval numbers are the evaluations of credit rating; the linguistic labels are the evaluation values of contractual capacity of partner and controlling system of financial risk. Because of the uncertainty information, the interval numbers provided by credit rating agency can be transformed into triangular fuzzy numbers, and the linguistic labels obtained by decision makers can be transformed into neutrosophic numbers. Therefore we can get the evaluation matrix *R* = (*r*_*ij*_) with crisp numbers, triangular fuzzy numbers and neutrosophic numbers.

Step 1 Aggregate financial data over several years.

The evaluation values of financial risk indexes such as asset liability ratio, current ratio, quick ratio, number of times interest earned, main business cost ratio, operating expense ratio, main business revenue growth rate, total asset growth rate, net assets yield, net profit growth rate, shareholder’s equity growth rate, equity ratio, retention ratio, cash debt coverage ratio, cash ratio and security surplus cash multiples are obtained by financial data over the years of company. In consideration of that the financial risk of a company influenced by historical financial condition, the historical data and current data which reflect development trend should be taken into account. In this section, according to the method introduced in Section 3.2, we aggregate financial data over the years by using exponential smoothing method through EViews software to get scientific and reasonable evaluation of financial risk. Then, the evaluation matrix of *R* = (*r*_*ij*_) based on *a*_11_, *a*_12_, *a*_13_, *a*_14_, *a*_21_, *a*_22_, *a*_23_, *a*_24_, *a*_25_, *a*_26_, *a*_31_, *a*_32_, *a*_33_, *a*_41_, *a*_42_, *a*_43_ is computed.

Step 2 Obtain the depiction of credit rating.

The evaluation information of credit rating is determined by credit rating agency. Because of the uncertainty and fuzziness of credit rating, it should be transformed into triangular fuzzy numbers. According to the method mentioned in Section 3.4, the relative descriptions and function of crediting rating can be conducted as seen in Table 4 and S3 Fig. Therefore, the evaluation matrix of *R* = (*r*_*ij*_) based on *a*_*15*_ is calculated.

**Table 4:** Fuzzy set of credit rating.

Credit rating	Credit value	Membership function	Fuzzy number
B	[0–50]	(0,25,50)	(0,0.25,0.5)
BB	[50–60]	(50,55,60)	(0.5,0.55,0.6)
BBB	[60–70]	(60,65,79)	(0.6,0.65,0.7)
A	[70–80]	(70,75,80)	(0.7,0.75,0.8)
AA	[80–90]	(80,85,90)	(0.8,0.85,0.9)
AAA	[90–100]	(90,95,100)	(0.9,0.95,1)

Step 3 Evaluate contractual capacity of partner and financial risk control system.

The fundamental thesis of neutrosophy presented in the study by Rivieccio [13] is that every idea has not only a certain degree of truth, as is generally assumed in many-valued logic contexts, but also a falsity degree and an indeterminacy degree that have to be considered independently from each other. As mentioned in [51] and [52], they can deal with consistent, hesitant, and inconsistent information at the same time, and benefit the management of the evaluation information mentioned in [53]. The evaluation values of contractual capacity of partner and financial risk control system are linguistic values determined by decision makers, so it must first be transformed into neutrosophic numbers with positive, medium and passive values [54]. In this section, we transform the linguistic evaluation information of contractual capacity of partner and financial risk control system according to the symmetric linguistic evaluation scale into neutrosophic numbers with truth, indeterminacy and falsity. Then, we can aggregate the neutrosophic numbers using single valued neutrosophic weighted averaging (SVNWA) aggregation operator described by formula (10). Therefore, the evaluation matrix of *R* = (*r*_*ij*_) based on *a*_*27*_ and *a*_*28*_ is calculated eventually, where *Ψ* = (*Ψ*_1_, *Ψ*_2_, …, *Ψ*_*p*_)^*T*^ is the weight vector of decision makers corresponding to these indexes.

### The calculation of index weight

In this section, the principle of the combination between subjectivity and objectivity is applied in the calculation of the index weight. First, we compute the entropy weights of financial risk indexes. Then, we can get comprehensive index weight which combines entropy weight of index with subjective weight of financial risk criteria.

Step 1 Determine the entropy weight of data index.

Normalizing the evaluation matrix in formula (19), and further normalization matrix *R* = (*r*_*ij*_)_p*q_ is ${\left[p_{ij}\right]}_{p\times q}={\left[r_{ij}/\sum_{i=1}^{p}r_{ij}\right]}_{p\times q}$ (*p* evaluation indicators, *q* evaluated objects). The normalization formula is as follows:

$$\begin{array}{ll} Z_{ij}=\frac{y_{ij}-y_{j}^{\text{min}}}{y_{j}^{\text{max}}-y_{j}^{\text{min}}} & if\;j\;is\;benefit\;index\\ Z_{ij}=\frac{y_{j}^{\text{max}}-y_{ij}}{y_{j}^{\text{max}}-y_{j}^{\text{min}}} & if\;j\;is\;cost\;index \end{array}.$$ (19)

The entropy weight can be defined as:

$$\begin{array}{l} E_{ij}=(1-e_{i})/\sum_{i=1}^{p}\left(p-e_{i}\right)\\ where\;e_{i}=-k\sum_{j=1}^{q}p_{ij}\;\text{ln}\;p_{ij},\;\;k=1/\text{ln}\;q\;,\;\sum_{i=1}^{p}E_{i}=1 \end{array}.$$ (20)

Hence, the entropy weight *E*_11_, *E*_12_, *E*_13_, *E*_14_, *E*_21_, *E*_22_, *E*_23_, *E*_24_, *E*_25_, *E*_26_, *E*_31_, *E*_32_, *E*_33_, *E*_41_, *E*_42_ and *E*_43_ can be calculated.

Step 2 Compute the entropy weight of credit rating index.

To normalize the TrFN, we use the following formula:

$$\begin{array}{l} x_{ij}=\{\begin{array}{ll} (a_{ij}/c_{i\text{max}},b_{ij}/c_{i\text{max}},c_{ij}/c_{i\text{max}}), & if\;i\;is\;benefit\;index\\ (1-c_{ij}/c_{i\text{max}},1-b_{ij}/c_{i\text{max}},1-a_{ij}/c_{i\text{max}}), & if\;i\;is\;\text{cos}t\;index \end{array}\\ where\;c_{i\text{max}}=\text{max}\{c_{ij}|j=1,2,\ldots ,n\} \end{array}.$$ (21)

Hence, the entropy weight *E*_15_ can be calculated by using formula (22).

Hi=−13lnn∑x=abc∑j=1nxij/(∑i=1pxij)lnxij/(∑i=1pxij)hi=Hi/∑i=1nHiE=ij(1−hi)/∑i=1p(1−hi). (22)

Step 3 Compute the entropy weight of contractual capacity of partner and controlling system of financial risk.

The following formula is used to normalize the neutrosophic numbers:

$$r_{ij}=\{\begin{array}{ll} T_{ij},I_{ij},F_{ij} & if\;j\;is\;benefit\;index\\ 1-T_{ij},1-I_{ij},1-F_{ij} & if\;j\;is\;\text{cos}t\;index \end{array}.$$ (23)

The formula (8) and formula (9) in Section 3.3 is used to calculate entropy weight based on evaluation matrix. Hence, the entropy weight *E*_27_ and *E*_28_ can be computed.

Step 4 Calculate financial risk index weight.

According to the explanation of BWM in Section 4.2, the optimal weight vector of financial risk criteria is $w_{i}^{*}$. The synthetic weight of financial risk index is calculated as the following way:

$$W_{ij}=w_{i}\times E_{ij}.$$ (24)

### The ranking order of companies according to financial risk by using TOPSIS

Suppose there are *n* alternatives *x*_*j*_ (*j* = 1, …, *n*), thus the sets of alternatives (i.e. companies) can be denoted by *X* = {*x*_1_, *x*_2_, …, *x*_*n*_}. The TOPSIS method based on heterogeneous MCDM is used to solve the ranking order of companies according to financial risk. Because of the existence of heterogeneous evaluation information of financial risk, the criteria set *A* = (*A*_1_, *A*_2_, *A*_3_, *A*_4_) can be divided into three subsets *O*_*i*_ (*i* = 1,2,3), where *O*_*i*_ are sets of criteria whose values are crisp numbers, triangular fuzzy numbers and neutrosophic numbers. The procedure of this method is summarized [17] as follows:

Step 1 Normalize evaluation matrix *R*.

The normalized evaluation value has already been solved at the time of entropy weight of risk index calculating. So we can get the normalized evaluation matrix *R* = (*r*_*ij*_) directly.

Step 2 Define the positive ideal solution (PIS) and the negative ideal solution (NIS) for each index.

Let *y*^+^ represents the PIS, *y*^-^ represents the NIS, where

$$y_{i}^{+}=\{\begin{array}{ll} e_{i}^{+} & ,if\;A_{i}\in o_{1}\\ \left(a_{i}^{+},b_{i}^{+},c_{i}^{+}\right) & ,if\;A_{i}\in o_{2}\\ \langle T_{i}^{+},I_{i}^{+},F_{i}^{+}\rangle & ,if\;A_{i}\in o_{3} \end{array}.$$ (25)

Here, $e_{i}^{+}=\text{max}\{e_{ij}|j=1,\ldots ,n\}\left(A_{i}\in o_{1}^{b}\right)$ or $\text{min}\{e_{ij}|j=1,\ldots ,n\}\left(A_{i}\in o_{1}^{c}\right)$; $(a_{i}^{+},b_{i}^{+},c_{i}^{+})=\text{max}\{a_{ij},b_{ij},c_{ij}|j=1,2,\ldots ,n\}\left(A_{i}\in o_{2}^{b}\right)$ or $\text{min}\{a_{ij},b_{ij},c_{ij}|j=1,2,\ldots ,n\}\left(A_{i}\in o_{2}^{c}\right)$; $\langle T_{i}^{+},I_{i}^{+},F_{i}^{+}\rangle \;=\langle \text{max}T_{ij},\text{min}I_{ij},\text{min}F_{ij}\rangle \left(A_{i}\in o_{3}^{b}\right)$ or $\langle \text{min}T_{ij},\text{max}I_{ij},\text{max}F_{ij}\rangle \left(A_{i}\in o_{3}^{c}\right)$.

Similarly, the NIS *y*^-^ is as follows:

$$y_{i}^{\text{-}}=\{\begin{array}{ll} e_{i}^{\text{-}} & ,if\;A_{i}\in o_{1}\\ \left(a_{i}^{\text{-}},b_{i}^{\text{-}},c_{i}^{\text{-}}\right) & ,if\;A_{i}\in o_{2}\\ \langle T_{i}^{\text{-}},I_{i}^{\text{-}},F_{i}^{\text{-}}\rangle & ,if\;A_{i}\in o_{3} \end{array}.$$ (26)

Here, $e_{i}^{-}=\text{min}\{e_{ij}|j=1,\ldots ,n\}\left(A_{i}\in o_{1}^{b}\right)$ or $\text{max}\{e_{ij}|j=1,\ldots ,n\}\left(A_{i}\in o_{1}^{c}\right)$; $(a_{i}^{-},b_{i}^{-},c_{i}^{-})=\text{min}\{a_{ij},b_{ij},c_{ij}|j=1,2,\ldots ,n\}\left(A_{i}\in o_{2}^{b}\right)$ or $\text{max}\{a_{ij},b_{ij},c_{ij}|j=1,2,\ldots ,n\}\left(A_{i}\in o_{2}^{c}\right)$; $\langle T_{i}^{-},I_{i}^{-},F_{i}^{-}\rangle \;=\langle \text{min}T_{ij},\text{max}I_{ij},\text{max}F_{ij}\rangle \left(A_{i}\in o_{3}^{b}\right)$ or $\langle \text{max}T_{ij},\text{min}I_{ij},\text{min}F_{ij}\rangle \left(A_{i}\in o_{3}^{c}\right)$.

Step 3 Compute the separation measures between each company and the PIS as well as the NIS.

The distance between the normalized values of the company *x*_*j*_ (*j* = 1, 2, …, *n*) and PIS *x*^+^ on all index *a*_*i*_ ∈ *o*_*1*_ is defined as follows.

$$\begin{array}{l} \rho (r_{o_{1}j},r_{o_{1}}^{+})=\sum_{a_{i}\in o_{1}}[W_{i}d(r_{ij},r_{i}^{+}){]}^{2}\\ \underset{a_{i}\in o_{1}}{d(r_{ij},r_{i}^{+})}=\left|e_{i}^{+}\text{-}e_{ij}\right| \end{array},$$ (27)

where $r_{o_{1}j}$ and $r_{o_{1}}^{+}$ are the normalized value vectors of the company *x*_*j*_ and the PIS *x*^+^ on all indexes in *O*_1_, respectively.

The distance between the normalized values of the company *x*_*j*_ (*j* = 1, 2, …, *n*) and PIS *x*^+^ on all indexes *a*_*i*_ ∈ *o*_2_ is described using formula (12) as follows.

$$\begin{array}{ll} \rho (r_{o_{2}j},r_{o_{2}}^{+}) & =\sum_{a_{i}\in o_{2}}[W_{i}d(r_{ij},r_{i}^{+}){]}^{2}\\ \underset{a_{i}\in o_{2}}{d}(r_{ij},r_{i}^{+}) & =\sqrt{\left(1/3\right)\left[{\left(a_{i}^{+}-a_{ij}\right)}^{2}+{\left(b_{i}^{+}-b_{ij}\right)}^{2}+{\left(c_{i}^{+}-c_{ij}\right)}^{2}\right]} \end{array},$$ (28)

where $r_{o_{2}j}$ and $r_{o_{2}}^{+}$ are the normalized value vectors of the company *x*_*j*_ and the PIS *x*^+^ on all indexes in *O*_2_, respectively.

The distance between the normalized values of the company *x*_*j*_ (*j* = 1, 2, …, *n*) and PIS *x*^+^ on all indexes *a*_*i*_ ∈ *o*_3_ is described using formula (7) as follows.

$$\begin{array}{ll} \rho (r_{o_{3}j},r_{o_{3}}^{+}) & =\sum_{a_{i}\in o_{3}}[W_{i}d(r_{ij},r_{i}^{+}){]}^{2}\\ \underset{a_{i}\in o_{3}}{d}(r_{ij},r_{i}^{+}) & =\;\sqrt{(1/3)\left[{\left(T_{i}^{+}-T_{ij}\right)}^{2}+{\left(I_{i}^{+}-I_{ij}\right)}^{2}+{\left(F_{i}^{+}-F_{ij}\right)}^{2}\right]} \end{array},$$ (29)

where $r_{o_{3}j}$ and $r_{o_{3}}^{+}$ are the normalized value vectors of the company *x*_*j*_ and the PIS *x*^+^ on all indexes in *O*_3_, respectively.

According to formula (27), (28), (29), the distance between a company *x*_*j*_ (*j* = 1, 2, …, *n*) and the PIS *x*^+^ is defined as follows:

$$\rho \left(r_{j},r^{+}\right)=\sqrt{\sum_{t=1}^{3}\rho (r_{o_{t}j},r_{o_{t}}^{+})}\;.$$ (30)

In the same way, the distance between the normalized values of the company *x*_*j*_ (*j* = 1, 2, …, *n*) and NIS *x*^-^ on all indexes *a*_*i*_ ∈ *o*_1_ is defined as follows:

$$\begin{array}{l} \rho (r_{o_{1}j},r_{o_{1}}^{\text{-}})=\sum_{a_{i}\in o_{1}}[W_{i}d(r_{ij},r_{i}^{\text{-}}){]}^{2}\\ \underset{a_{i}\in o_{1}}{d(r_{ij},r_{i}^{\text{-}})}=\left|e_{ij}\text{-}e_{i}^{-}\right| \end{array},$$ (31)

where $r_{o_{1}}^{\text{-}}$ is the normalized value vector of the NIS *x*^-^ on all indices in *O*_1_.

The distance between the normalized values of the company *x*_*j*_ (*j* = 1, 2, …, *n*) and NIS *x*^-^ on all indices *a*_*i*_ ∈ *o*_2_ is expressed as follows:

$$\begin{array}{l} \rho (r_{o_{2}j},r_{o_{2}}^{\text{-}})=\sum_{a_{i}\in o_{2}}[W_{i}d(r_{ij},r_{i}^{\text{-}}){]}^{2}\\ \underset{a_{i}\in o_{2}}{d}(r_{ij},r_{i}^{\text{-}})=\sqrt{\left(1/3\right)\left[{\left(a_{i}^{\text{-}}-a_{ij}\right)}^{2}+{\left(b_{i}^{\text{-}}-b_{ij}\right)}^{2}+{\left(c_{i}^{\text{-}}-c_{ij}\right)}^{2}\right]} \end{array}$$ (32)

Where $r_{o_{2}}^{\text{-}}$ is the normalized value vector of the NIS *x*^-^ on all indices in *O*_2_.

The distance between the normalized values of the company *x*_*j*_ (*j* = 1, 2, …, *n*) and NIS *x*^-^ on all indexes *a*_*i*_ ∈ *o*_3_ is introduced as follows.

$$\begin{array}{l} \rho (r_{o_{3}j},r_{o_{3}}^{\text{-}})=\sum_{a_{i}\in o_{3}}[W_{i}d(r_{ij},r_{i}^{\text{-}}){]}^{2}\\ \underset{a_{i}\in o_{3}}{d}(r_{ij},r_{i}^{\text{-}})=\;\sqrt{(1/3)\left[{\left(T_{i}^{\text{-}}-T_{ij}\right)}^{2}+{\left(I_{i}^{\text{-}}-I_{ij}\right)}^{2}+{\left(F_{i}^{\text{-}}-F_{ij}\right)}^{2}\right]} \end{array},$$ (33)

where $r_{o_{3}}^{\text{-}}$ is the normalized value vector of the NIS *x*^-^ on all indices in *O*_3_.

Using the formula (31), (32), (33), the distance between a company *x*_*j*_ (*j* = 1, 2, …, *n*) and the NIS *x*^-^ is defined as follows:

$$\rho \left(r_{j},r^{\text{-}}\right)=\sqrt{\sum_{t=1}^{3}\rho (r_{o_{t}j},r_{o_{t}}^{\text{-}})}.$$ (34)

Step 4 Calculate relative closeness degree of companies to the PIS.

$$\tau_{i}=\frac{\rho \left(r_{j},r^{\text{-}}\right)}{\rho \left(r_{j},r^{+}\right)\text{+}\rho \left(r_{j},r^{\text{-}}\right)}.$$ (35)

Step 5 Rank the companies according to *τ*_*i*_.

The bigger *τ*_*i*_ is, the better company *x*_*j*_ will be.

## Empirical study

### Background and data collection

At present, biological medicine is one of the most important emerging industries in China. And the financial risk assessment is conducive to the risk control and healthy development of pharmaceutical companies. In this section, we selected three companies from Chinese A-share pharmaceutical manufacturing listed companies randomly and conducted an empirical study in order to verify the effectiveness of the proposed model. The stock codes of the three companies are 600196, 600664 and 600085, denoted by *A*, *B*, *C* respectively.

The original financial data and related information can be collected from the website http://www.qianzhan.com or credit rating agency, and the subjective information can be obtained from supervisors of company through questionnaire surveys. In the study, supervisors from the three companies were invited to participate in a questionnaire to obtain linguistic assessment. The raw data on the operation and technology of the three companies from 2010 to 2016, the original evaluation information of credit rating, contractual capacity of partner and financial risk control system were collected, as supporting information; see S1 Raw Data.

According to the financial risk assessment model proposed in Section 4, we compute financial risk criteria weight and get the evaluation matrix through historical financial information of company, credit rating agency and decision makers. Then, the synthetic weight of financial risk index is computed by multiplying criteria weight with the entropy weight of risk index. Finally, the ranking order of the three companies according to their financial risk can be calculated using TOPSIS method based on heterogeneous MCDM. In addition, the effectiveness and reliability of the proposed financial risk assessment model are verified by comparative analysis and sensitivity analysis.

### Financial risk criteria weight

According to the BWM method introduced in Section 4.2, we calculate the subjective weight of financial risk criteria. Among the financial risk criteria denoted by *A*_*1*_ to *A*_*4*_, listed in Table 2, financing risk (*A*_1_) is the most important criterion, and income distribution risk (*A*_3_) is the least important criterion, it is determined by experts. The pairwise comparison vector of the most important and least important criteria can be described in Tables 5 and 6. Table 5 indicates that the preference values of the most important criterion (*A*_1_) over criterion (*A*_2_), criterion (*A*_3_) and criterion (*A*_4_) are 3, 8 and 6 respectively. The preference values of the criterion (*A*_1_), (*A*_2_) and (*A*_4_) over the least important criterion (*A*_3_) are 8, 7 and 5, respectively, which are shown in Table 6. Therefore, the weight vector of criteria $w^{*}=\left(w_{1}^{*},w_{2}^{*},w_{3}^{*},w_{4}^{*}\right)$ is computed.

**Table 5:** The pairwise comparison vector of the most important criterion.

Criteria	*A*_1_	*A*_2_	*A*_3_	*A*_4_
Most important criterion: A_1_	1	3	8	6

**Table 6:** The pairwise comparison vector of the least important criterion.

Criteria	*A*_1_	*A*_2_	*A*_3_	*A*_4_
Least important criterion: *A*_3_	8	7	1	5

From formula (17) and (18) illustrated in Rezaei’s study [36], we can get *w*_1_* = 0.3809, *w*_2_* = 0.3334, *w*_3_* = 0.0476, *w*_4_* = 0.2381, and *ξ*^L*^ = 0. Based on the proposed method, *ξ*^L*^ indicates consistency index directly without extra computation. As *ξ*^L*^ = 0, we can obtain complete consistency. Thus, the subjective criteria weight is w* = (0.3809, 0.3334, 0.0476, 0.2381).

### Evaluation matrix

According to the evaluation method proposed in Section 4.3, the evaluation matrix determined by historical financial information of company, credit rating agency and decision makers is obtained, and the information is heterogeneous, including crisp numbers, interval numbers and linguistic labels.

Firstly, the evaluation matrix of numerical index is calculated by aggregating the historical financial data of 2010–2016, according to the exponential smoothing forecasting method introduced in Section 4.3. And the final evaluation values of financial risk on quantitative indices are listed in Table 7. The data visualization is depicted as shown in S4–S7 Figs.

**Table 7:** Evaluation values of financial risk on quantitative indices.

Company	600196	600664	600085
Index *a*_*ij*_			
Asset liability ratio *a*_*11*_	0.433078	0.452117	0.332912
Current ratio *a*_*12*_	1.351786	1.637926	3.067071
Quick ratio *a*_*13*_	1.099651	1.081613	1.566228
Number of times interest earned *a*_*14*_	7.699711	-27.2296	-52.1203
Main business cost ratio *a*_*21*_	0.54996	0.713979	0.56649
Operating expense ratio *a*_*22*_	0.384665	0.223827	0.2724
Main business revenue growth rate *a*_*23*_	0.129798	0.089747	0.134532
Total asset growth rate *a*_*24*_	0.274039	-0.06922	0.201173
Net assets yield *a*_*25*_	0.083663	0.401227	0.083086
Net profit growth rate *a*_*26*_	0.219956	0.135309	0.196705
Shareholder’s equity growth rate *a*_*31*_	0.497309	0.525481	0.474622
Equity ratio *a*_*32*_	0.23558	0.105562	0.194374
Retention ratio *a*_*33*_	4.19662	12.77456	2.717608
Cash debt coverage ratio *a*_*41*_	0.30631	0.324754	1.086585
Cash ratio *a*_*42*_	0.866545	0.355547	1.315133
Security surplus cash multiples *a*_*43*_	1.981559	5.262483	3.913923

Secondly, we compute the evaluation value of credit rating.

According to the evaluation method of credit rating introduced in Section 4.3, we translate the evaluation information of credit rating into triangular fuzzy numbers transformed by membership function, as shown in Table 8.

**Table 8:** Evaluation values of credit rating *a*_*15*_.

Company	600196	600664	600085
Fuzzy number	(0.9,0.95,1)	(0.7,0.75,0.8)	(0.8,0.85,0.9)

Thirdly, we get the assessment value of contractual capacity of partner and financial risk control system.

Considering the deficiency of practical information and the efficiency of neutrosophic set mentioned in [55], we transform decision makers’ linguistic labels into neutrosophic numbers by using the method introduced in Section 4.3, and aggregate the neutrosophic numbers using SVNWA operator described in formula (10). The description of evaluation values is shown in Table 9.

**Table 9:** Evaluation values of financial risk by decision makers.

Indexes	Company		
	600196	600664	600085
*a*_*27*_	<0.86285,0,0>	<0.67489,0,0>	<0.77567,0,0>
*a*_*28*_	<0.85465,0,0>	<0.53128,0,0>	<0.74035,0,0>

Therefore, the evaluation matrix *R* = (*r*_*ij*_) can be determined directly, as shown in Tables 7, 8 and 9.

### Weight of financial risk index

The weight of the indexes can be calculated by using the entropy weight method introduced in Section 4.4. According to the normalization method, the normalized evaluation matrix is described in Table 10.

**Table 10:** Normalized evaluation matrix.

index	600196	600664	600085
*r*_*11*_	0.159716	0	1
*r*_*12*_	0	0.166818	1
*r*_*13*_	0.037221	0	1
*r*_*14*_	1	0.416094	0
*r*_*15*_	(0.9,0.95,1)	(0.7,0.75,0.8)	(0.8,0.85,0.9)
*r*_*21*_	1	0	0.899219
*r*_*22*_	0	1	0.698
*r*_*23*_	0.894295	0	1
*r*_*24*_	1	0	0.78772
*r*_*25*_	0.001814	1	0
*r*_*26*_	1	0	0.725318
*r*_*27*_	<0.86285,0,0>	<0.67489,0,0>	<0.77567,0,0>
*r*_*28*_	<0.85465,0,0>	<0.53128,0,0>	<0.74035,0,0>
*r*_*31*_	0.446076	1	0
*r*_*32*_	1	0	0.683075
*r*_*33*_	0.147064	1	0
*r*_*41*_	0	0.023638	1
*r*_*42*_	0.532519	0	1
*r*_*43*_	0	1	0.588969

The entropy weight is computed by formula (20), formula (22) and formula (9), the result is obtained as *E*_1_ = (*E*_11_, *E*_12_, *E*_13_, *E*_14_, *E*_15_) = (0.196258983, 0.193583809, 0.265492452, 0.138674702, 0.20599005); *E*_2_ = (*E*_21_, *E*_22_, *E*_23_, *E*_24_, *E*_25_, *E*_26_, *E*_27_, *E*_28_) = (0.085170054, 0.088203688, 0.085201275, 0.086354441, 0.227198836, 0.08753941, 0.177338538, 0.162993756); *E*_3_ = (*E*_31_, *E*_32_, *E*_33_) = (0.296810103, 0.261347451, 0.441842446); *E*_4_ = (*E*_41_, *E*_42_, *E*_43_) = (0.525714023, 0.240714367, 0.233571609). The eventual weights of financial risk indexes are calculated by synthesizing subjective and objective weights, which is calculated by formula (24). The result is described as *E*_1_ = (*E*_11_, *E*_12_, *E*_13_, *E*_14_, *E*_15_) = (0.074755047, 0.073736073, 0.101126075, 0.052821194, 0.078461612); *E*_2_ = (*E*_21_, *E*_22_, *E*_23_, *E*_24_, *E*_25_, *E*_26_, *E*_27_, *E*_28_) = (0.028395696, 0.02940711, 0.028406105, 0.028790571, 0.075748092, 0.029185639, 0.059124669, 0.054342118); *E*_3_ = (*E*_31_, *E*_32_, *E*_33_) = (0.014128161, 0.012440139, 0.0210317); *E*_4_ = (*E*_41_, *E*_42_, *E*_43_) = (0.125172509, 0.057314091, 0.0556134). Thus, according to the description in Section 4.4, the weight of the attributes and indexes are obtained, as in Table 11.

**Table 11:** The weight of the attributes and indexes.

Index	*E*_*11*_	*E*_*12*_	*E*_*13*_	*E*_*14*_	*E*_*15*_	*E*_*21*_	*E*_*22*_	*E*_*23*_	*E*_*24*_	*E*_*25*_
Weight	0.074755	0.073736	0.101126	0.052821	0.078462	0.028396	0.029407	0.028406	0.028791	0.075748
Index	*E*_*26*_	*E*_*27*_	*E*_*28*_	*E*_*31*_	*E*_*32*_	*E*_*33*_	*E*_*41*_	*E*_*42*_	*E*_*43*_	
Weight	0.029186	0.059125	0.054342	0.014128	0.01244	0.021032	0.125173	0.057314	0.055613	

### Ordering result of TOPSIS method

Based on the method illustrated in Section 4.5, the degrees of similarity with respect to the PIS are calculated for the three companies *x*_1_, *x*_2_, *x*_3_ as follows:

$$\rho \left(r_{1},r^{\text{-}}\right)=0.11090758$$

$$\rho \left(r_{1},r^{+}\right)=0.04531486$$

$$\tau_{1}=\frac{\rho \left(r_{1},r^{\text{-}}\right)}{\rho \left(r_{1},r^{+}\right)\text{+}\rho \left(r_{1},r^{\text{-}}\right)}\;=0.709933734$$

$$\rho \left(r_{2},r^{\text{-}}\right)=0.010980454$$

$$\rho \left(r_{2},r^{+}\right)=0.069108298$$

$$\tau_{2}=\frac{\rho \left(r_{2},r^{\text{-}}\right)}{\rho \left(r_{2},r^{+}\right)\text{+}\rho \left(r_{2},r^{\text{-}}\right)}\;=0.137103576$$

$$\rho \left(r_{3},r^{\text{-}}\right)=0.050895848$$

$$\rho \left(r_{3},r^{+}\right)=0.016607356$$

$$\tau_{3}=\frac{\rho \left(r_{3},r^{\text{-}}\right)}{\rho \left(r_{3},r^{+}\right)\text{+}\rho \left(r_{3},r^{\text{-}}\right)}\;=0.753976767.$$

It is easy to conclude the following ranking order of the three companies: *x*_3_ ≻ *x*_1_ ≻ *x*_2_. Therefore, the company with the lowest financial risk is *x*_3_, i.e. 600085.

### Comparison analysis and discussion

As described in Section 4, the proposed model can be used to assess the financial risk of company considering historical financial information, credit rating, the conditions of partners and risk control, and heterogeneous information. To validate that the proposed model can effectively and reliably identify which company has the lowest financial risk, a comparative analysis is made with heterogeneous TODIM (an acronym in Portuguese of interactive and multi-criteria decision making) [56] and heterogeneous VIKOR (VlseKriterijumska Optimizacija I Kompromisno Resenje) [57] in the empirical study. Table 12 shows the ranking order of the three companies as obtained using these methods. Based on Table 12, the ranking order calculated by the proposed hybrid assessment model are the same as those computed by heterogeneous TODIM method and heterogeneous VIKOR method, so the effectiveness of the model is proved. Compared with other assessment methods of financial risk for companies, the advantages of the proposed model in the paper can be generalized as the following:

**Table 12:** Ranking comparison.

Alternatives	Heterogeneous TODIM		Heterogeneous VIKOR			
	*ε*_*i*_	Ranking	*S*_*i*_	*R*_*i*_	*Q*_*i*_	Ranking
*x*_*1*_	0.165428173	2	0.575278	0.125172509	0.801890377	2
*x*_*2*_	0	3	0.766834	0.122213681	0.970067145	3
*x*_*3*_	1	1	0.283375	0.075748092	0	1

1. The proposed model considers both subjective and objective indexes in the financial risk index system, and combines fuzzy theory with quantitative data analysis. Thus effectively ensuring that the financial risk assessment for companies can be more in line with reality.
2. The evaluation information is evaluated from historical financial data of the company, credit rating agency and decision-makers, including crisp numbers, triangular fuzzy numbers and neutrosophic numbers. So that the financial risk assessment model is more accurate and reliable.
3. In the proposed model, TOPSIS method is used to determine the ranking order of financial risk of the companies, which is more flexible and simple in solving MGCDM problem [16]. Therefore, the proposed financial risk assessment model for companies can obtain the best company with the least financial risk reliably.

### Sensitivity analysis

In order to monitor the robustness of the financial risk assessment model for companies, the sensitivity analysis is conducted according to the change of the weight coefficient *∂* and the evaluator’s attitude *λ*. The corresponding ranking order of the three companies can be obtained when the value of *∂* is changed, which are listed in Table 13. And the influence on the proposed financial risk assessment model with different values of *∂* listed in Table 13 can be figured in S8 Fig.

**Table 13:** Ranking order of companies with different *∂*.

Different *∂*	The ranking of companies			Ranking order
	*x*_*1*_	*x*_*2*_	*x*_*3*_	
*∂* = 0.2	0.745634776	0.165568264	0.765469954	*x*_2_ ≺ *x*_1_ ≺ *x*_3_
*∂* = 0.4	0.745141545	0.148393814	0.749196829	*x*_2_ ≺ *x*_1_ ≺ *x*_3_
*∂* = 0.5	0.743693347	0.221579234	0.746581992	*x*_2_ ≺ *x*_1_ ≺ *x*_3_
*∂* = 0.6	0.72784475	0.213714928	0.757485834	*x*_2_ ≺ *x*_1_ ≺ *x*_3_
*∂* = 0.8	0.710154635	0.231682333	0.740883248	*x*_2_ ≺ *x*_1_ ≺ *x*_3_

According to Table 13 and S8 Fig, it is clear that the ranking order calculated are the same as in the above experimental example, when the value of *∂* is changed from 0 to 1. This means that the ranking order is insensitive to the changes of parameter *∂*. That is to say, despite the assessment process involving different values of the weighting coefficient *∂*, the final ranking order is consistent.

When the evaluator’s attitude *λ* is changed, the evaluation values of credit rating are transformed into triangular fuzzy number, the influence of values *λ* on the proposed model is shown in Table 14 and S9 Fig. Obviously, the changes of the evaluator’s attitude *λ* do not influence the ranking order of the three companies, and the results are the same as that of the above experimental example.

**Table 14:** Ranking order of companies with different *λ*.

Different *λ*	The ranking of companies			Ranking order
	*x*_*1*_	*X*_*2*_	*x*_*3*_	
*λ* = 0.2	0.709933734	0.137103576	0.753976767	*x*_2_ ≺ *x*_1_ ≺ *x*_3_
*λ* = 0.4	0.709933734	0.137103576	0.753976767	*x*_2_ ≺ *x*_1_ ≺ *x*_3_
*λ* = 0.5	0.709933734	0.137103576	0.753976767	*x*_2_ ≺ *x*_1_ ≺ *x*_3_
*λ* = 0.6	0.709933734	0.137103576	0.753976767	*x*_2_ ≺ *x*_1_ ≺ *x*_3_
*λ* = 0.8	0.709933734	0.137103576	0.753976767	*x*_2_ ≺ *x*_1_ ≺ *x*_3_

According to the visualized results shown in S8 and S9 Figs, the final ranking order is consistent in the experimental example of the sensitivity analysis. In other words, although the different selection of weighting coefficient *∂* and evaluator’s attitude *λ*, *x*_*3*_ is the best company with the least financial risk. The two sensitivity analysis results indicate that the ranking order of the proposed model is insensitive to the values of *∂* and *λ* in the example. Therefore, to a certain degree, the robustness of the proposed model is verified.

## Conclusion and future research

In this paper, a multi-level fuzzy comprehensive financial risk assessment model for companies has been developed. In order to assess the company’s financial risk accurately, subjective and objective indexes have been utilized simultaneously in the financial risk index system, which combines fuzzy theory with quantitative data analysis. Moreover, heterogeneous information obtained from historical financial information of company, credit rating agency and the decision makers’ estimation, such as crisp numbers, triangular fuzzy numbers and neutrosophic numbers, has been employed to decrease the information loss. In addition, TOPSIS based on heterogeneous MCDM has been employed to obtain the ranking order of companies according to their financial risk.

The proposed model has been used in empirically study to assess the financial risks of the listed pharmaceutical manufacturing companies of Chinese A-share. Moreover, the comparison results with the two other methods show that the proposed model is effective and reliable. In addition, the sensitivity analysis has been carried out and the results verify the robustness of the proposed model.

In summary, this paper not only contributes to the development of theory, but also contributes to practical application. First, the proposed model uses both quantitative historical data analysis and fuzzy theory; it is helpful to enrich the contents of risk research. Second, the proposed model will optimize the financial risk assessment method for companies. Third, the proposed model can be applied to provide rational support for decision makers in the process of financial risk management.

There are several possible directions of further research. First, more information types of financial risk assessment could be considered in the proposed model in order to adapt to the dynamic financial environment in future research. Second, the proposed model can obtain the ranking order of companies according to financial risk by investigating MULTIMOORA (multi–objective optimization by ratio analysis plus the full multiplicative form) method because of its simple computation. Finally, the proposed model can be adopted for risk assessment for some other fields in future study.

## Supporting information

S1 Fig Summary of the process of the proposed model. (TIF)

S2 Fig Financial risk index system. (TIF)

S3 Fig The membership function of credit rating. (TIF)

S4 Fig Financing risk. (TIFF)

S5 Fig Investment risk. (TIF)

S6 Fig Income distribution risk. (TIF)

S7 Fig Cash flow at risk. (TIFF)

S8 Fig The result of the sensitivity analysis with different *∂*. (TIF)

S9 Fig The radar plot displaying the result of the sensitivity analysis. (TIFF)

S1 Raw Data (PDF)
