# Supplementary figures and images for: A novel financial risk assessment model for companies based on heterogeneous information and aggregated historical data
Source: PLoS One. 2018 Dec 26;13(12):e0208166. doi: 10.1371/journal.pone.0208166 (PMC6306178; doi:10.1371/journal.pone.0208166)

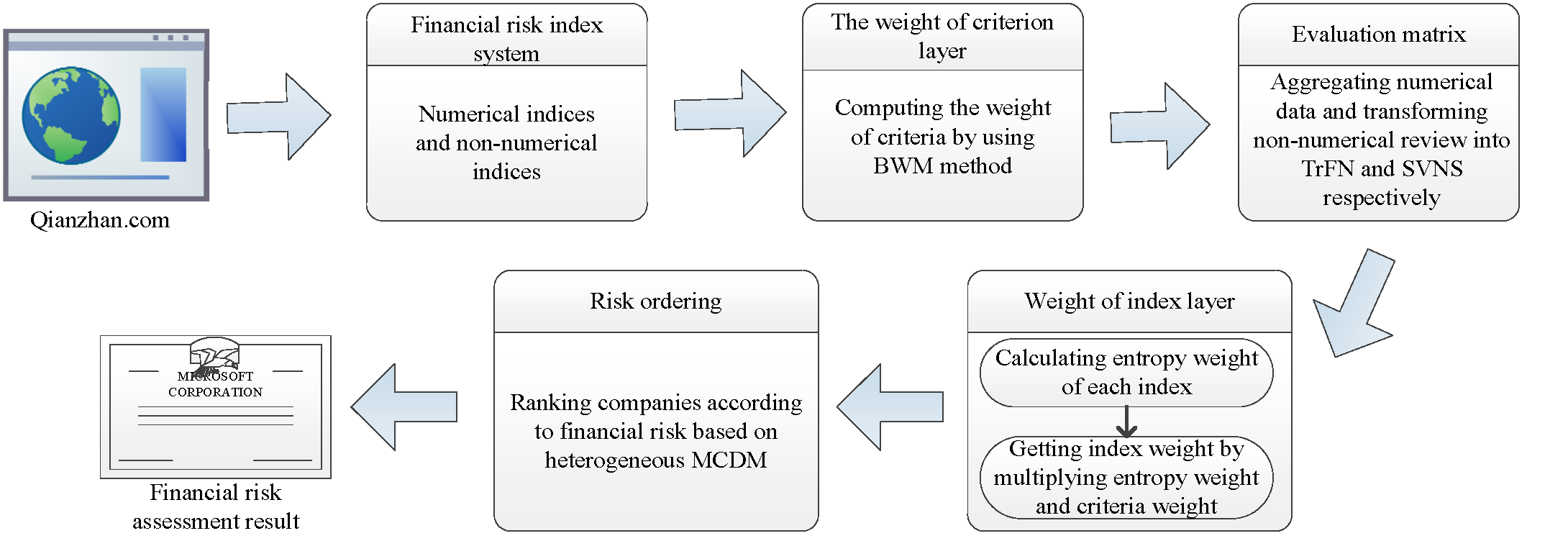

Supplement: S1 Fig — (TIF) [file pone.0208166.s001.tif]

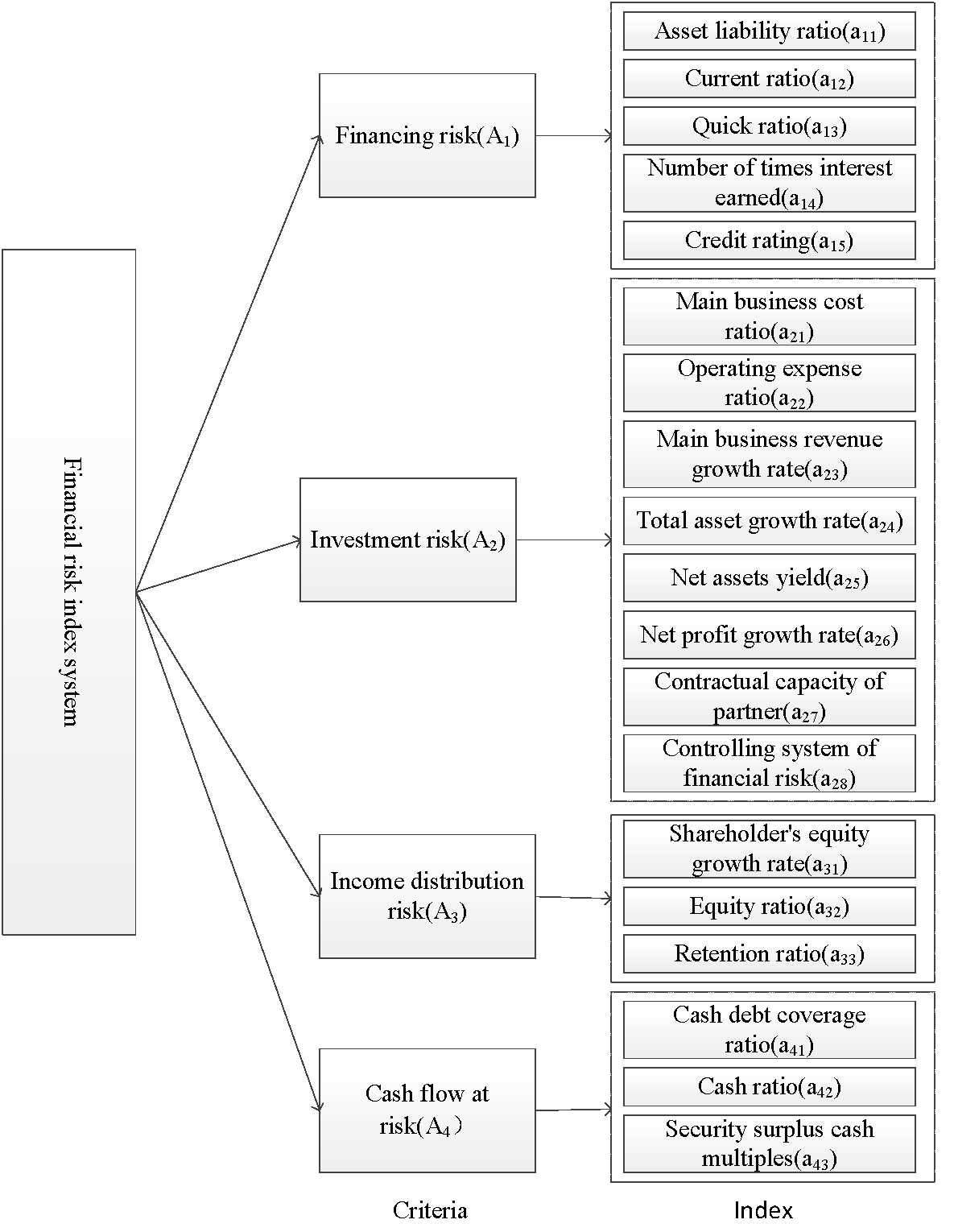

Supplement: S2 Fig — (TIF) [file pone.0208166.s002.tif]

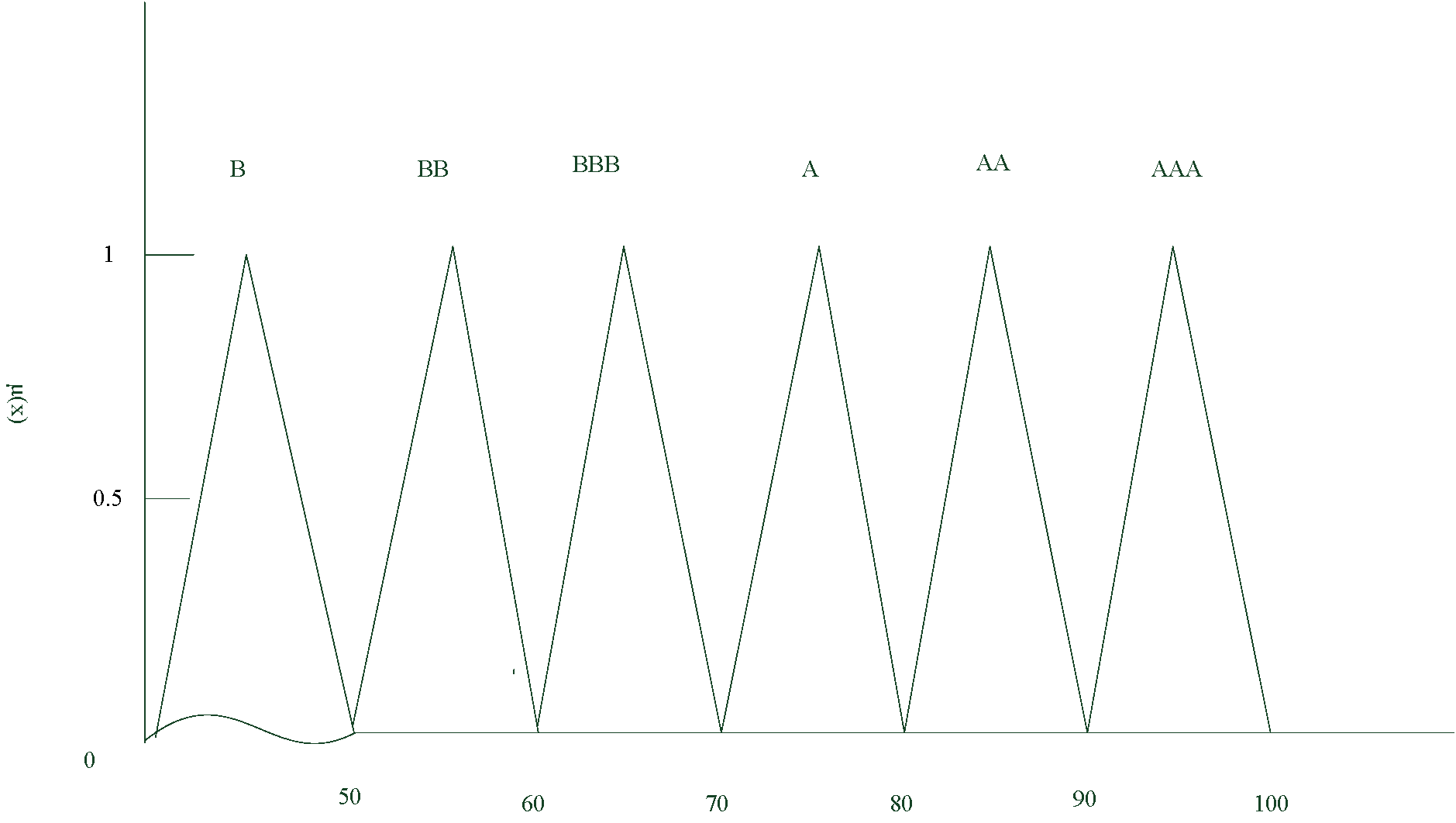

Supplement: S3 Fig — (TIF) [file pone.0208166.s003.tif]

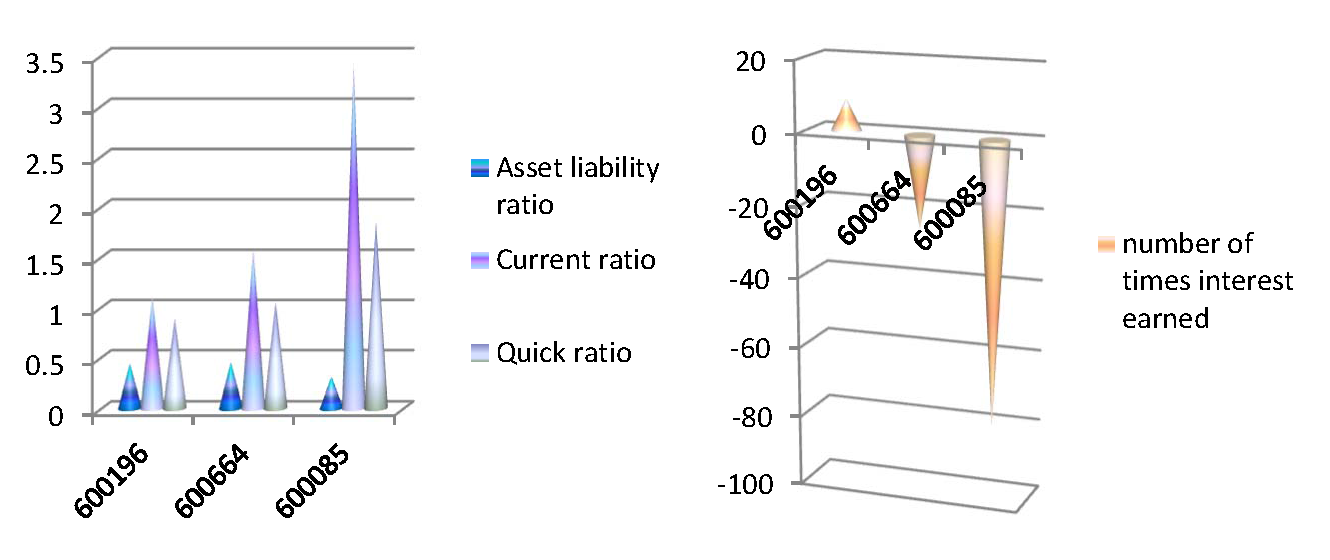

Supplement: S4 Fig — (TIFF) [file pone.0208166.s004.tiff]

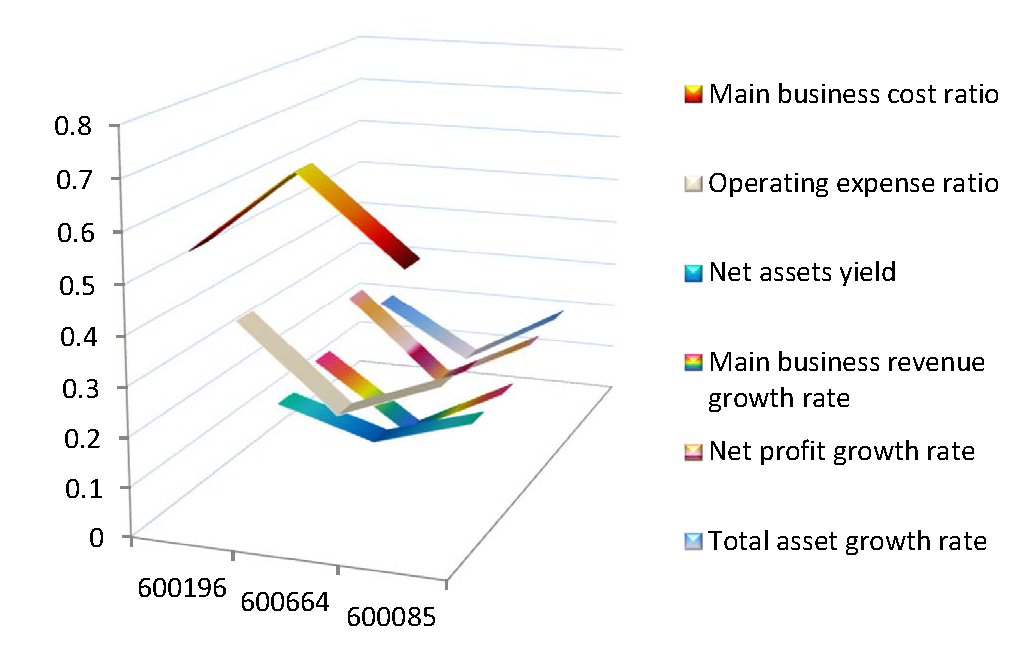

Supplement: S5 Fig — (TIF) [file pone.0208166.s005.tif]

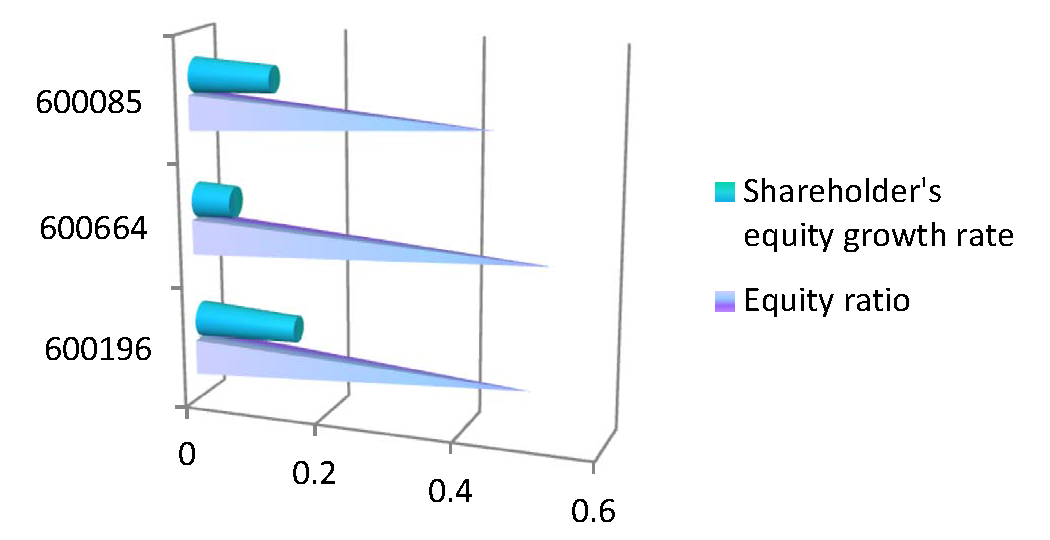

Supplement: S6 Fig — (TIF) [file pone.0208166.s006.tif]

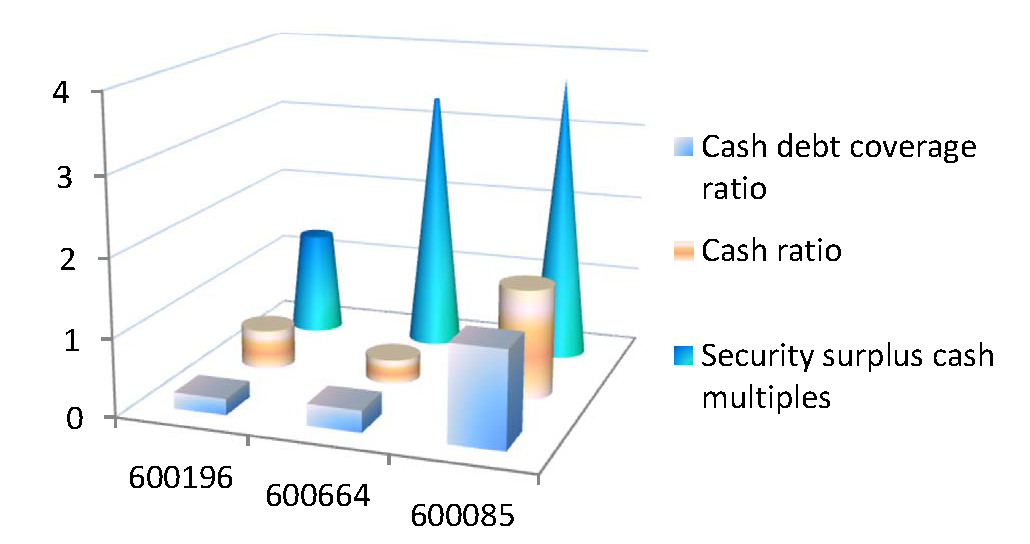

Supplement: S7 Fig — (TIFF) [file pone.0208166.s007.tiff]

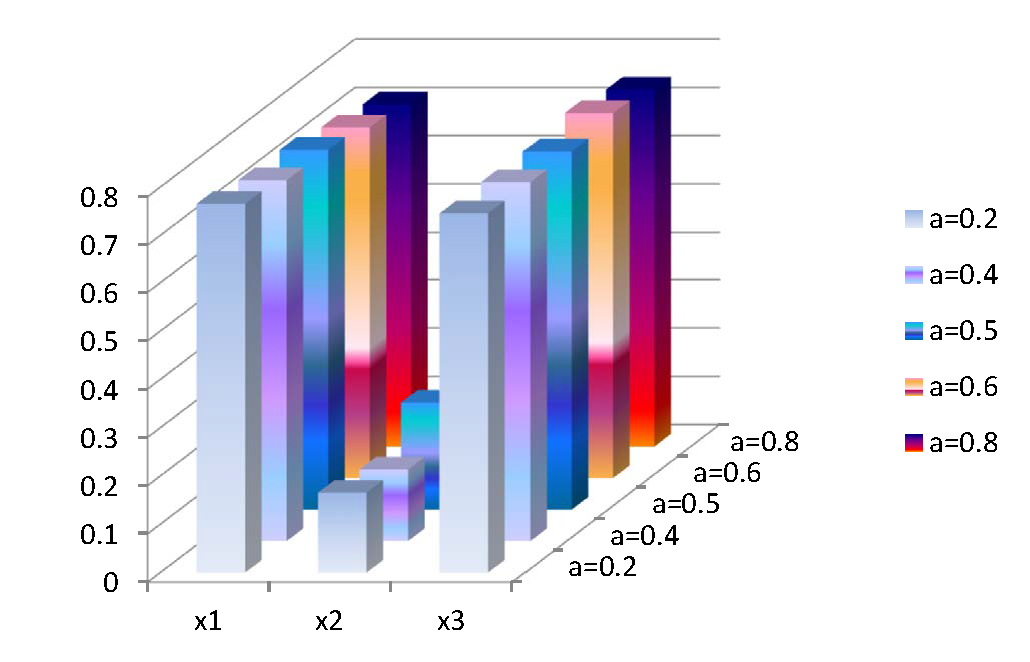

Supplement: S8 Fig — (TIF) [file pone.0208166.s008.tif]

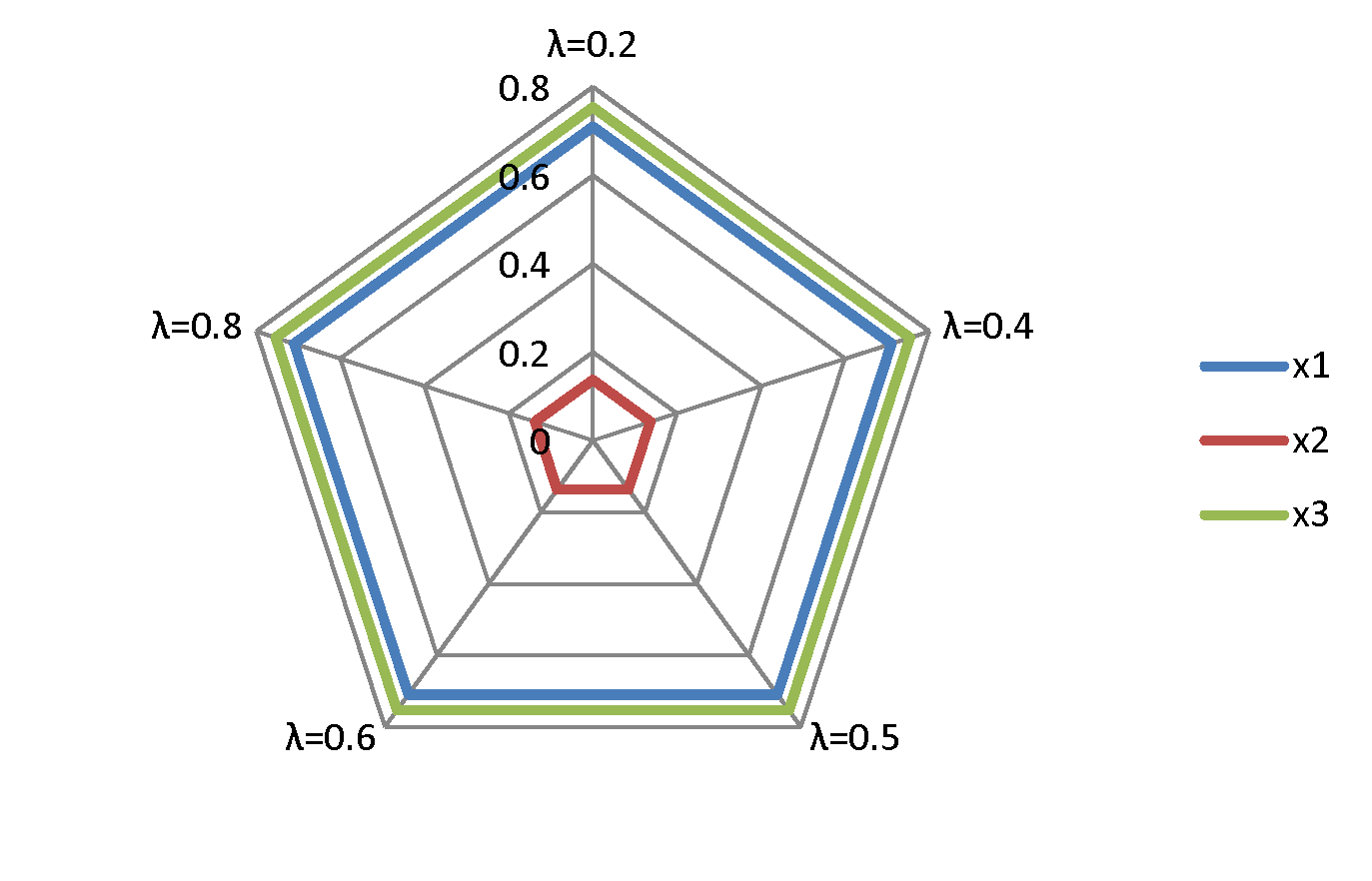

Supplement: S9 Fig — (TIFF) [file pone.0208166.s009.tiff]
