# Supplementary material for: A novel financial risk assessment model for companies based on heterogeneous information and aggregated historical data
Source: PLoS One. 2018 Dec 26;13(12):e0208166. doi: 10.1371/journal.pone.0208166 (PMC6306178; doi:10.1371/journal.pone.0208166)
Supplement: S1 Raw Data — (PDF) [file pone.0208166.s010.pdf]

Table A$_1$. Original data of financing risk.

Stock code	Year	Asset liability ratio	Current ratio	Quick ratio	Number of times interest earned
600196	2010	0.4415	1.6	1.31	8.39
	2011	0.4899	1.21	0.94	6.96
	2012	0.4	2.15	1.76	6.74
	2013	0.4007	1.32	0.97	8.58
	2014	0.4594	0.91	0.72	8.18
	2015	0.4589	0.76	0.59	8.49
	2016	0.4231	1.06	0.87	9.91
600664	2010	0.4435	1.65	1.09	-56.31
	2011	0.4499	1.62	1.06	-12.8
	2012	0.4478	1.7	1.11	-32.76
	2013	0.4669	1.59	1.06	-7.78
	2014	0.4601	1.56	1.02	-6.43
	2015	0.4564	1.49	1.05	-21.62
	2016	0.4487	1.66	1.33	-36.02
600085	2010	0.231	3.63	1.88	-83.2
	2011	0.3436	2.46	1.03	-294.36
	2012	0.4203	2.79	1.45	127.34
	2013	0.3421	3.35	1.87	49.87
	2014	0.3321	3.27	1.76	-151.28
	2015	0.2419	3.44	1.82	-106.79
	2016	0.292	3.43	1.97	-102

Table A$_2$. Original data of investment risk.

Stock code	Year	Main business cost ratio	Operating expense ratio	Net assets yield	Main business revenue	Net profit growth rate	Total asset growth rate

					growth rate		
600196	2010	0.655166678	0.33596422	0.1159	0.1764	-0.6543	0.4592
	2011	0.615391487	0.36783372	0.1281	0.4237	0.3496	0.3252
	2012	0.562174903	0.39883673	0.1341	0.1319	0.3417	0.1443
	2013	0.554536034	0.36490226	0.1403	0.3618	0.2961	0.1556
	2014	0.558692023	0.36638952	0.132	0.203	0.0423	0.1988
	2015	0.500294762	0.41010731	0.1412	0.0485	0.1643	0.0811
	2016	0.459255348	0.43864464	0.139	0.1602	0.1405	0.1457
600664	2010	0.701712947	0.196457	0.181	0.174	0.2115	0.1754
	2011	0.765610426	0.178749	0.0898	0.0759	-0.488	-0.0088
	2012	0.681275161	0.257122	0.0697	0.3096	-0.1365	0.3438
	2013	0.704503667	0.265891	0.0209	0.0243	-0.6624	0.0503
	2014	0.724773325	0.236549	0.0302	-0.0875	0.465	-0.0129
	2015	0.750716328	0.183308	0.0759	-0.0395	1.3477	-0.1525
	2016	0.735603076	0.17948	0.1057	-0.1091	0.3585	0.0935
600085	2010	0.545874	0.279184	0.1091	0.18	0.20	0.12
	2011	0.590036	0.257449	0.1301	0.60	0.28	0.34
	2012	0.560877	0.278281	0.1529	0.23	0.30	0.32
	2013	0.571288	0.273895	0.1458	0.16	0.15	0.23
	2014	0.568277	0.266484	0.145	0.11	0.16	0.09
	2015	0.539208	0.283223	0.1385	0.12	0.15	0.11
	2016	0.540223	0.28659	0.1252	0.12	0.07	0.19

Table A_3. Original data of income distribution risk.

Stock code	Year	Equity ratio	Shareholder's equity growth rate	Retention ratio
600196	2010	0.5008	0.351152048	4.691246549
	2011	0.4384	0.210531855	4.092903764
	2012	0.5316	0.34595731	3.828420709

	2013	0.5202	0.154192748	3.582604849
	2014	0.4719	0.081433754	3.004178132
	2015	0.4759	0.082010498	3.111613636
	2016	0.507	0.2216137	3.450529838
600664	2010	0.5535	0.093782	3.829868
	2011	0.5468	-0.02019	6.578959
	2012	0.5078	0.349072	8.599988
	2013	0.4953	0.013993	38.40169
	2014	0.5072	-0.00035	26.96849
	2015	0.5123	-0.14676	6.549596
	2016	0.5217	0.108927	5.703625
600085	2010	0.5933	0.078797	4.181502
	2011	0.4743	0.139716	2.987484
	2012	0.4115	0.164895	2.652481
	2013	0.4213	0.398442	2.494495
	2014	0.4263	0.10323	2.521857
	2015	0.4964	0.257207	2.549848
	2016	0.4568	0.111122	2.789918

Table A$_4$. Original data of cash flow risk.

Stock code	Year	Cash debt coverage ratio	Cash ratio	Security surplus cash multiples
600196	2010	0.400050667	0.961872	2.970109776
	2011	0.222364614	0.626215	1.753426219
	2012	0.408878017	1.334973	2.268058141
	2013	0.204582723	0.58953	1.006796939
	2014	0.185431179	0.391039	1.270194105
	2015	0.190998105	0.368275	1.1664887
	2016	0.245067967	0.593164	1.408741103

600664	2010	0.476758453	0.510273	2.195911
	2011	0.299205412	0.326051	2.700489
	2012	0.28749469	0.318484	3.418131
	2013	0.223148843	0.252335	12.55614
	2014	0.309394397	0.345812	11.69599
	2015	0.28602575	0.325304	2.924591
	2016	0.682900355	0.763748	5.466663
600085	2010	1.411777	1.474243	3.800068
	2011	0.825377	0.859093	3.175727
	2012	0.895785	1.255714	4.142962
	2013	1.199672	1.654597	4.582408
	2014	1.067972	1.429277	3.662392
	2015	1.363329	1.4304	3.228487
	2016	1.166546	1.494207	3.720026

Table B_1. Credit rating

Company	Credit rating	Interval
600196	AAA	90-100
600664	A	70-80
600085	AA	80-90

Table B_2. Evaluation values of contractual capacity of partner.

Company	Neutrosophic number			
600196	<0.95,0,0>	<0.847,0,0>	<0.815,0,0>	<0.75,0,0>
600664	<0.535,0,0>	<0.8,0,0>	<0.79,0,0>	<0.428,0,0>
600085	<0.621,0,0>	<0.791,0,0>	<0.784,0,0>	<0.852,0,0>

Table B_3. Evaluation values of financial risk control system.

Company	Neutrosophic number

600196	<0.852,0,0>	<0.886,0,0>	<0.818,0,0>
600664	<0.525,0,0>	<0.428,0,0>	<0.621,0,0>
600085	<0.791,0,0>	<0.779,0,0>	<0.621,0,0>
